# Isolation of peripheral blood mononuclear cells and the expression of toll-like receptors in Betong chickens

**DOI:** 10.14202/vetworld.2020.1372-1375

**Published:** 2020-07-18

**Authors:** Anutian Suklek, Autchara Kayan, Jatuporn Rattanasrisomporn, Chaiwat Boonkaewwan

**Affiliations:** 1 Department of Animal Science, Faculty of Agriculture, Kasetsart University, Bangkok 10900, Thailand; 2 Department of Companion Animal Clinical Science, Faculty of Veterinary Medicine, Kasetsart University, Bangkok 10900, Thailand; 3 Akkhraratchakumari Veterinary College, Walailak University, Nakhon Si Thammarat 80161, Thailand

**Keywords:** Betong chicken, peripheral blood mononuclear cell, toll-like receptor

## Abstract

**Background and Aim:**

Toll-like receptors (TLRs) comprise microbial sensing receptors present on cell surfaces that are capable of detecting pathogens. The present study aims to examine the expression of TLRs within the peripheral blood mononuclear cell (PBMC) of the Betong chickens.

**Materials and Methods:**

Blood samples were harvested from 12 Betong (KU line) chickens. Hematological values were calculated. PBMC was isolated from the blood utilizing a Histopaque solution and stored in a RPMI1640 culture medium. Cell viability was investigated using a Trypan Blue dye exclusion test. DNA was extracted from PBMC and the expression of the DNA’s TLRs was examined using a polymerase chain reaction.

**Results:**

Hematological values were determined from the blood samples collected in this study obtained from healthy Betong chickens. PBMC that was isolated from the Betong chickens possessed cell viability higher than 95% (95.37±1.06). From the examination of TLRs gene expression, results revealed instances of TLR1.1, TLR1.2, TLR2.1, TLR2.2, TLR3, TLR4, TLR5, TLR 7, TLR15, and TLR21 that were present in the PBMC of Betong chickens.

**Conclusion:**

PBMC isolated from the blood of healthy Betong chickens possessed excellent cell quality. All chicken TLRs were discovered within the PBMC of Betong chickens. Hence, PBMC stands out as one of the premier sources for *in vitro* studies of chicken immune response.

## Introduction

Toll-like receptors (TLRs) belong to the pattern recognition receptor system, which detects pathogens through their recognizable molecular patterns, which include multiple components of pathogens including lipopolysaccharides, peptidoglycans, flagellin, bacterial DNA, and viral double-stranded RNA [1]. TLRs orchestrate a vital role within the innate immune response, and TLRs are expressed within a wide scope of tissues and cell types [2]. Ten chicken TLR types, including TLR1.1, TLR1.2, TLR2.1, TLR2.2, TLR3, TLR4, TLR5, TLR 7, TLR15, and TLR21, have been identified [3,4]. TLRs characteristics were detected in several types of chicken tissues. The majority of tissues contained at least one TLR occurrence; however, many tissues displayed numerous instances of TLRs present therein [5,6].

Peripheral blood mononuclear cells (PBMCs) consist of a mixed population of single nuclear white blood cells, which are comprised heterogeneous population of blood cells, including monocyte and lymphocyte immune cells. The latter is a category containing natural killer cells, B-cells, and T-cells. PBMCs play an integral role in innate and adaptive immune responses due to their ability to recognize and evade pathogens [7]. Hence, PBMC is most likely the strongest source for the assessment of differences or changes within the gene expression associated with disease or pathogens’ responses.

It has previously been widely established that differing chicken breeds vary in their resistance or susceptibility to diseases. The variation across chicken breeds in immune response during the course of infection is attributed to the variation in the chicken breed’s respective disease susceptibility [8]. TLR profile expression is also known to vary among various chicken types [9]. To further examine and improve the health of Betong chickens, the present study aimed to investigate the prevalence of the TLRs gene within PBMCs.

## Materials and Methods

### Ethical approval

All procedures used in this study were approved by the Animal Ethics Committee of Kasetsart University (ACKU61-AGR-009).

### Animal and blood collection

Twelve Betong chickens aged 14-18 weeks old sourced from the Vajokkasikij Chicken farm located at Kasetsart University were utilized within the present study. Blood samples (3 mL) were harvested through the wing vein of the chickens and then placed in EDTA-containing tubes.

### Hematology

Blood (0.5 ml) was utilized for the purpose of conducting a hematological study. The total red and white blood cell counts were examined manually utilizing a hemocytometer. Varying white blood count profiles were carried out on monolayer blood films, fixed, and tested using a Giemsa-Wright’s stain. Hematocrit was manually measured utilizing microhematocrit capillary tubes and then centrifuged at 2.500 rpm for 5 min. Hemoglobin concentration (Hb) was also measured using the cyanmethemoglobin method.

### PBMC isolation and cell viability test

PBMCs were separated from blood samples following the method described by Böyum [10]. Briefly, 2.5 mL of blood was gently layered over 2 mL of Histopaque solution (Sigma-Aldrich, St. Louis, MO), then centrifuged at 1500 rpm for 30 min. The white band of mononuclear cells was collected and washed 3 times using a RPMI 1640 culture medium through centrifugation at 3000 rpm for 5 min. PBMCs were suspended within a RPMI 1640 culture medium (containing 25 mM HEPES and 2 mM L-glutamine) and then adjusted to 2 × 10^6^ cells/ml. Cell viability assays were investigated using a Trypan Blue dye exclusion test.

### DNA extraction

Genomic DNA was prepared from the harvested PBMC utilizing DNA extraction kits (biotechrabbit, Germany) adhering to the manufacturer’s instruction. DNA sample concentrations were calculated using spectrophotometry at the wavelength of 260 nm, and the purity was observed utilizing OD 260/OD 280, in NanoDrop equipment (Biodrop, UK).

### Polymerase chain reactions (PCR)

PCRs were performed for TLR1.1, TLR1.2, TLR2.1, TLR2.2, TLR3, TLR4, TLR5, TLR7, TLR15, TLR21, and β-actin following standard protocols (Green PCR Master Mix, biotechrabbit, Germany), with the primers displayed in Table-1 [5]. The β-actin gene was chosen to confirm the quality of genomic DNA samples. Briefly, 13.5 μL reaction volumes contained 1 μL of genomic DNA, 1.25 μL of each primer, 5 μL of ddH2O, and 6.25 μL of Green Master Mix. The cycling conditions included initial denaturation at 95°C for 5 min followed by 35 cycles at 95°C for 1 min. The optimum annealing temperature of each TLR was 58.5-60.5°C (Table-1), and optimal annealing time was 25 s, and the final extension step was 10 min at 72°C. Negative control was present consisting of the PCR lacking genomic DNA from each sample. PCR products were stored at 4°C. A 10 μl of each PCR product was subsequently electrophoresed on 1.5% agarose gel at 100 V for 15 min, and DNA bands were visualized using ethidium bromide under UV light (Gel Doc XR System, Qiagen).

**Table-1:** Sequence of primers and annealing temperature used in PCR.

Target gene	Primer (5’-3’)	Accession no.	Annealing temp. (°C)
TLR-1.1	F:AGGTGGGACTTCTTATTGAGGCATAC R:AGATGAATCCCAAACTAGCAGAAAAA	AJ633574	58
TLR-1.2	F: AGTCCATCTTTGTGTTGTCGCC R: AACGCTGCTTTCAAGTTTTCCC	NM_001081709	58.5
TLR2.1	F: ACATGTGTGAATGGCCTGAA R: TTGAGAAATGGCAGTTGCAG	NM_204278	58.5
TLR-2.2	F: AGGCACTTGAGATGGAGCAC R: CCTGTTATGGGCCAGGTTTA	AB046533	58
TLR3	F: AGACACAGCAATTCAGAAC R: TTAATGATGTTATTATCCTCCAAG	NM_001011691	59
TLR4	F: TGCACAGGACAGAACATCTCTGGA R: AGCTCCTGCAGGGTATTCAAGTGT	AY064697	59.5
TLR5	F: CACTGCTGGAGGATTTGTTCTTG R: ACAGACGGAGTATGGTCAAACG	NM_001024586	59.5
TLR7	F: GATGCAGTGTGGTTTGTTGG R: AACCAAGCTCCTCCCTTTGT	NM_001011688	59
TLR15	F: TCTTCTGGTATCTGGTCTTGC R: CCTGGATTGGGTGGATCTTC	NM_001037835	59
TLR21	F: AGCTGGAGCTGTTGGACCTA R: TTCACGTGCCATAGCATCTC	NM_001030558	59.5
beta-actin	F: GCACCACACTTTCTACAATAG R: ACGACCAGAGGCATACAGG	L08165	60.5

## Results

### Hematological values

To confirm the blood samples collected from healthy Betong chickens, the complete blood count was examined. The hematological values are displayed in Table-2. Total erythrocyte count hemoglobin and hematocrit were 2.33±0.28 cell/mm^3^, 8.76±1.00 g/dl, and 26.00±3.33%, respectively. The total white blood cell and differential leukocyte counts of heterophil, eosinophil, basophil, lymphocyte, and monocyte were 7874.17±2505.52 cell/mm^3^, 66.33±8.85%, 0.25±0.62%, 1.92±1.83%, 26.83±9.00, and 3.42±1.08%, respectively.

**Table-2:** Hematological values of Betong chicken (n=12).

Hematology	Betong (KU line)
Red blood cell (10^6^/μl)	2.33±0.28
Hemoglobin (g/dl)	8.76±1.00
Hematocrit (%)	26.00±3.33
White blood cell (cells/mm^3^)	7874.17±2505.52
Heterophil (%)	66.33±8.85
Basophil (%) g	0.25±0.62
Eosinophil (%)	1.92±1.83
Lymphocyte (%)	26.83±9.00
Monocyte (%)	3.42±1.08

### PBMC cell viability

To deduce the amount of viable cells presented in PBMC suspension, cell viability findings are displayed in Table-3. Results demonstrated that the cell viability of PBMC isolated from Betong chickens ranged from 93.80% to 97.22%.

**Table-3:** Peripheral blood mononuclear cell viability (n=12).

No.	Cell viability (%)
1	94.40
2	93.80
3	95.55
4	94.79
5	94.47
6	96.31
7	97.22
8	95.03
9	95.72
10	94.66
11	97.01
12	95.47
Average	95.37±1.06

### Expression of TLRs

**Figure-1:**
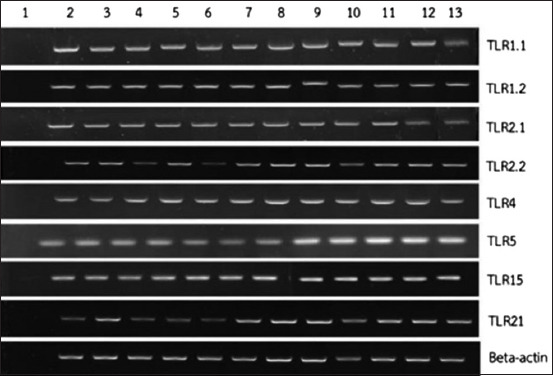
Toll-like receptor which responded to bacteria in peripheral blood mononuclear cells of Betong chickens. Lane 1 is negative control. Lanes 2-13 are 12 DNA samples of Betong chickens.

**Figure-2:**
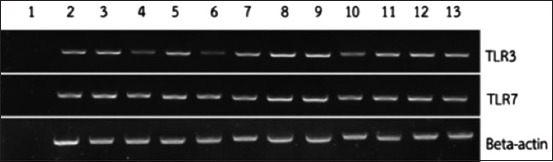
Toll-like receptor which responded to virus in peripheral blood mononuclear cells of Betong chickens. Lane 1 is negative control. Lanes 2-13 are 12 DNA samples of Betong chickens.

PCR method was used to characterize each gene of TLRs in PBMC which responded to bacteria. Results demonstrated that TLR1.1, TLR1.2, TLR2.1, TLR2.2, TLR4, TLR5, TLR15, and TLR21 expressions were present within the PBMC of Betong chicken (Figure-1). Furthermore, TLRs which responded to viruses were additionally identified and results revealed TLR3 and TLR7 expression present within the PBMC of Betong chickens (Figure-2).

## Discussion

The Betong chicken comprises a popular food type in the Southern region of Thailand due to strong meat quality, low carcass fat, and high levels of lean meat compared to other native Thai chicks. The Betong chicken possesses a much faster growth rate when compared to other native chickens. The Betong chicken also possesses other advantages, like being able to live in a hot climate well and the capability to thrive on low-quality feed. Recently, there has been an increase in demand for the Betong chicken from Thai people, especially in Bangkok [11,12]. The importance of hematological parameters as diagnostic tools and physiological indicators within chickens has been well documented. Although the reference values of avian hematological indices have been recorded, only a handful of studies on hematology values for the local Thai native chickens have been published to date. The values of all examined hematological parameters in the present study were within the same reference range as previously reported [13,14]. Hematological values disclosed that blood samples in this study were obtained from healthy Betong chickens.

The Trypan Blue dye exclusion test is employed to determine the amount of viable cells present in a cell suspension. The test is based on the principle that live cells possess intact cell membranes that exclude certain dyes, whereas dead cells do not and let the dye in. Cell viability assessments provide an early indicator of the quality of fresh cells. Viabilities of greater than or equal to 95% are considered excellent [15]. This study demonstrated that the average PBMC viability was >95%, underscoring the fact that PBMCs isolated from Betong chickens possess excellent cell quality.

TLRs comprise a major class of innate immune pattern recognition receptors that play an integral role in facilitating immune homeostasis and bolstering the body’s defense against infections. To date, 10 chicken TLRs including TLR1.1, TLR1.2, TLR2.1, TLR2.2, TLR3, TLR4, TLR5, TLR7, TLR15, and TLR21 have been identified [3,16]. The prevalence of the aforementioned TLRs indicators varied across several chicken tissue types [17-19]. Of note, an initial study revealed that all 10 chicken TLRs were present in the PBMC of Betong chickens. It follows that PBMC is one of the strongest sources for *in vitro* studies of chicken immune response. However, further studies are necessary to observe the level of mRNA expression of each TLR within the PBMC of healthy chickens, as TLR genes can serve as molecular markers in the rearing of future disease-resistant chickens [20,21].

## Conclusion

PBMC prepared from the blood samples of healthy Betong chickens had excellent cell quality. All chicken TLRs (TLR1.1, TLR1.2, TLR2.1, TLR2.2, TLR3, TLR4, TLR5, TLR7, TLR15, and TLR21) were found in the PBMC of Betong chickens. Therefore, PBMC is possibly one of the best sources for the study of chicken immune response.

## Authors’ Contributions

AS collected samples and performed experiments. AK and JR provided technical help during the experiments. CB designed the experiments, collected samples, and revised the manuscript. All authors read and approved the final manuscript.
